# Optical Coherence Tomography Angiography of Punctate Inner Choroidopathy

**DOI:** 10.1155/2017/4754231

**Published:** 2017-11-05

**Authors:** Blake M. Hampton, Christopher M. Aderman, Harry W. Flynn, Jayanth Sridhar

**Affiliations:** ^1^Bascom Palmer Eye Institute, University of Miami, Miami, FL, USA; ^2^Mid Atlantic Retina, The Retina Service of Wills Eye Hospital, Philadelphia, PA, USA

## Abstract

**Purpose:**

To report a case of bilateral choroidal neovascularization (CNV) in punctate inner choroidopathy (PIC) visualized utilizing optical coherence tomography angiography (OCT-A).

**Methods:**

Case report.

**Results:**

A 29-year-old woman presented with new visual symptoms in both eyes. Fundoscopic exam revealed bilateral multifocal, small, well-defined lesions consistent with PIC. Optical coherence tomography demonstrated subretinal fluid and retinal pigment epithelium detachments (RPEDs) in both eyes. OCT-A revealed bilateral abnormal increased flow within the RPEDs consistent with CNV. Fluorescein angiography confirmed the presence of bilateral CNV.

**Conclusion:**

CNV secondary to PIC may be identified using noninvasive optical coherence tomography angiography.

## 1. Introduction

Punctate inner choroidopathy (PIC) is a posterior uveitis belonging to the group of idiopathic white dot syndromes. It tends to present in young to middle aged women with myopia [1]. Choroidal neovascularization (CNV) may develop in the setting of PIC, leading to visual impairment [2]. Optical coherence tomography (OCT) and fluorescein angiography (FA) are most commonly utilized to identify and monitor CNV in patients with PIC.

Optical coherence tomography angiography (OCT-A) is a relatively new, noninvasive imaging modality that had been previously shown to successfully identify CNV in patients with diseases such as neovascular age-related macular degeneration (AMD) before a recently published series demonstrated its utility in PIC [3, 4]. This report highlights the usefulness of OCT-A in cases of PIC to guide management.

## 2. Case Presentation

**Figure 1:**
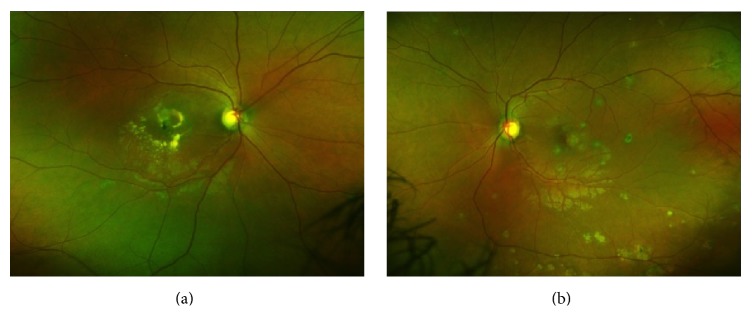
Fundus photography of the right (a) and left (b) eyes demonstrating multiple posterior small variably pigmented lesions consistent with punctate inner choroidopathy.

**Figure 2:**
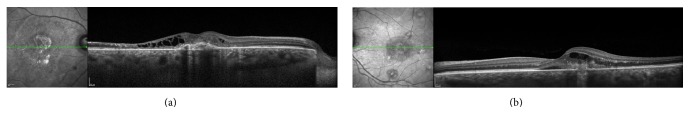
(a) Right eye optical coherence tomography revealing retinal pigment epithelium disruption and elevation and subretinal deposition with intraretinal cystic changes. (b) Left eye optical coherence tomography revealing retinal pigment epithelium disruption with associated subretinal and intraretinal fluid.

**Figure 3:**
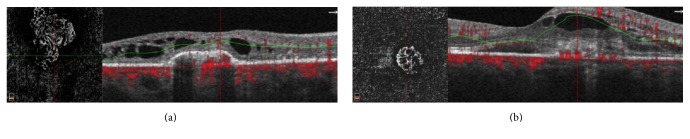
Optical coherence tomography angiography at level of retinal pigment epithelium elevation in right (a) and left (b) eyes demonstrates abnormal flow consistent with choroidal neovascularization.

**Figure 4:**
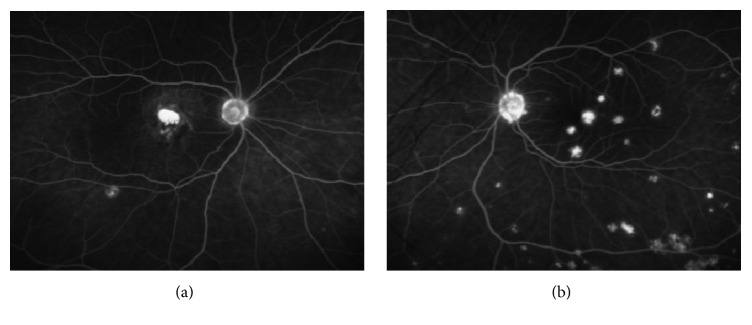
Fluorescein angiography of right (a) and left (b) eyes reveals late leakage posteriorly consistent with choroidal neovascularization.

A 29-year-old woman with no prior medical history presented with a new black spot in her right eye and wavy lines in her left eye. Best corrected Snellen visual acuity was finger count in the right eye and 20/40 in the left eye. Anterior chamber examination was unremarkable and trace vitreous cell was noted in both eyes. Fundoscopic exam revealed multiple, small, punched-out variably pigmented lesions in the posterior pole of both eyes with scattered peripheral lesions (Figure 1). OCT (Spectralis HRA + OCT, Heidelberg Engineering, Inc., Heidelberg, Germany) demonstrated RPE disruption and deposition with intraretinal fluid with retinal atrophy in the right eye (Figure 2(a)) and RPE disruption with subretinal and intraretinal fluid in the left eye (Figure 2(b)). OCT-A (RTVue-XR Avanti, Optovue, Fremont, CA) at the level of the RPE disruption in both eyes revealed increased abnormal flow consistent with bilateral CNV (Figure 3). FA (Optos, Optos Inc., Dunfermline, United Kingdom) demonstrated late leakage corresponding to the CNV (Figure 4). The patient received bilateral intravitreal injections of bevacizumab and was lost to follow-up after a single treatment.

## 3. Discussion

FA and OCT are the current gold standard for identifying and monitoring CNV. One limitation of these techniques is that CNV and inflammatory lesions may present similarly with elevation of the retinal pigment epithelium (RPE) and subretinal fluid on OCT and leakage on FA [5]. It is important to be able to differentiate the two, as CNV typically requires prompt treatment with intravitreal antivascular endothelial growth factor (anti-VEGF) agents whereas treatment of inflammatory lesions relies on immunosuppressant therapy [5]. A recently published case series demonstrated the value of OCT-A in detecting CNV in PIC. In some instances, FA was unable to distinguish active inflammation from CNV while OCT-A clearly revealed abnormal flow corresponding to CNV [4]. OCT-A carries the additional advantage of being noninvasive and avoiding potential rare side effects such as anaphylaxis from fluorescein dye.

As the technology continues to evolve, OCT-A should continue to provide more information about classically described disease processes such as PIC. In the same case series, OCT-A showed the progression of CNV activity as measured by flow while OCT imaging remained stable [4]. Beyond its ability to simply visualize CNV, in the future OCT-A may ultimately help elucidate how those new vessels respond to anti-VEGF treatment [6].
